# Treatment Efficiency Following Pediatric Tibial Lengthening Using Monolateral External Fixation: The Role of Surgical Planning and Postoperative Complications

**DOI:** 10.3390/jcm15166198

**Published:** 2026-08-11

**Authors:** Manuel A. Rodríguez-Cigala, Daniela Velázquez-Aréstegui, Silvestre Fuentes-Figueroa, Carlos A. Guzmán-Martín

**Affiliations:** 1 Shriners Hospitals Mexico, Mexico City 04600, Mexico; manuel_azael_rc@hotmail.com (M.A.R.-C.); fuentessilvestre@gmail.com (S.F.-F.); 2 Research Programs, Shriners Hospitals Mexico, Mexico City 04600, Mexico

**Keywords:** Pediatric tibial lengthening, monolateral external fixation, distraction osteogenesis, treatment efficiency, complication burden, healing index, surgical planning

## Abstract

**Background:** Pediatric tibial lengthening requires prolonged treatment and may be complicated by adverse events that affect both achievement of the planned correction and biological treatment efficiency. Evidence regarding these complementary dimensions in children treated with monolateral external fixation remains limited. **Methods:** We conducted a retrospective cohort study of pediatric patients who underwent tibial lengthening with a monolateral external fixator between June 2018 and June 2023 at a tertiary pediatric orthopedic center. Planned and achieved lengthening, treatment duration, distraction, maturation, and healing indices, and postoperative complications were evaluated. Complications were analyzed according to overall occurrence, cumulative burden, and pin-site severity. Associations were explored using nonparametric comparisons, Spearman correlations, logistic regression, and robust linear regression. **Results:** Thirty-nine patients were included; the median age was 14 years (interquartile range [IQR], 11–16), and 69.2% were male. Median distraction, maturation, and healing indices were 0.60 mm/day (IQR, 0.46–0.78), 4.48 days/mm (IQR, 3.03–6.50), and 6.29 days/mm (IQR, 4.90–9.00), respectively. Patients who failed to achieve the planned correction had greater planned lengthening targets. After adjustment for overall complication status, each additional millimeter of planned lengthening was associated with approximately 7% higher odds of failure to achieve the planned goal (odds ratio, 1.07; 95% confidence interval, 1.01–1.16; *p* = 0.046). Postoperative complications were associated with longer distraction and healing periods and higher healing indices. In robust models adjusted for achieved lengthening, any complication was associated with an approximately 41% higher healing index (*p* = 0.010), while each increase in pin-site severity was associated with an approximately 22% higher healing index (*p* = 0.009). Increasing complication burden showed a progressive pattern of prolonged healing. **Conclusions:** Pediatric tibial lengthening outcomes may be understood through two complementary dimensions. Greater planned lengthening was associated with a lower probability of fully achieving the intended correction, whereas postoperative complications primarily affected treatment efficiency and duration. Careful planning and early complication control may therefore represent complementary strategies for reducing treatment burden.

## 1. Introduction

Lower limb length discrepancy (LLD) in pediatric patients may result in altered gait, pelvic imbalance, and long-term functional impairment [1,2,3]. Distraction osteogenesis remains the cornerstone of surgical management, enabling controlled bone regeneration and soft-tissue adaptation under mechanical tension [4,5,6].

Circular external fixation remains a reference technique, although monolateral external fixators are increasingly used in pediatric patients. Their simpler construct, easier postoperative management, and improved patient comfort make them an attractive alternative [7,8,9]. Nevertheless, concerns remain regarding mechanical stability, alignment control, complication profiles, and bone consolidation dynamics compared with circular fixation systems [10].

Clinical outcomes following tibial lengthening are commonly assessed using distraction, maturation, and healing indices, which provide standardized measures of treatment efficiency throughout the different phases of distraction osteogenesis [11,12]. These indices may be influenced by patient characteristics, underlying etiology, surgical factors, and postoperative complications, all of which contribute to the biological response to limb lengthening [13,14]. Among these factors, complications such as pin-site infection, joint stiffness, and regeneration-related disorders remain major challenges because they may prolong treatment, increase patient burden, and compromise rehabilitation [15,16,17].

Despite the widespread use of tibial lengthening in pediatric limb reconstruction, the extent to which specific complication profiles influence treatment efficiency remains incompletely characterized, particularly in patients treated with monolateral external fixation. Although complications are frequently reported, their relationship with the temporal dynamics of distraction osteogenesis, including distraction, maturation, and healing indices, has not been comprehensively evaluated. A better understanding of these associations may improve patient counseling, perioperative decision-making, and postoperative management strategies while providing more realistic expectations regarding treatment duration and recovery [5,9,18]. Therefore, the aim of this study was to evaluate the relationship between complication profiles and lengthening efficiency, as measured by distraction, maturation, and healing indices, in pediatric patients undergoing tibial lengthening with monolateral external fixation.

## 2. Materials and Methods

### 2.1. Study Design

We conducted a retrospective observational cohort study involving consecutive pediatric patients who underwent tibial lengthening using the Rekrea^®^ monolateral external fixator (Citieffe, Calderara di Reno, Bologna, Italy) between June 2018 and June 2023 at Shriners Hospital for Children, Mexico City. Clinical and radiographic records were reviewed to evaluate treatment efficiency, bone healing, complication profiles, and achievement of planned lengthening goals.

All patients underwent routine preoperative evaluation, including medical history, physical examination, and standard laboratory assessment. None of the included patients had documented metabolic bone disease (e.g., osteogenesis imperfecta, rickets, renal osteodystrophy), endocrine disorders affecting skeletal growth, or syndromic skeletal dysplasias. Therefore, limb length discrepancy or tibial nonunion in this cohort was attributed to localized congenital deformities (hemimelia, femoral hypoplasia, congenital tibial pseudarthrosis) or post-traumatic etiologies rather than systemic metabolic pathology.

### 2.2. Data Collection

Demographic, clinical, radiographic, and surgical variables were extracted from institutional electronic medical records and standardized radiographic evaluations.

Baseline variables included age at surgery, sex, body mass index (BMI), etiological diagnosis, and limb length discrepancy. Surgical variables included planned lengthening, achieved lengthening, duration of distraction, maturation period, healing period, and total external fixation time.

To provide a comprehensive assessment of treatment efficiency, additional derived variables were calculated, including percentage of planned lengthening achieved and absolute lengthening shortfall (planned minus achieved lengthening).

### 2.3. Outcome Measures

The principal outcomes of interest were achievement of the planned lengthening goal and treatment efficiency.

### 2.4. Lengthening Indices and Operational Definitions

**Figure 1:**
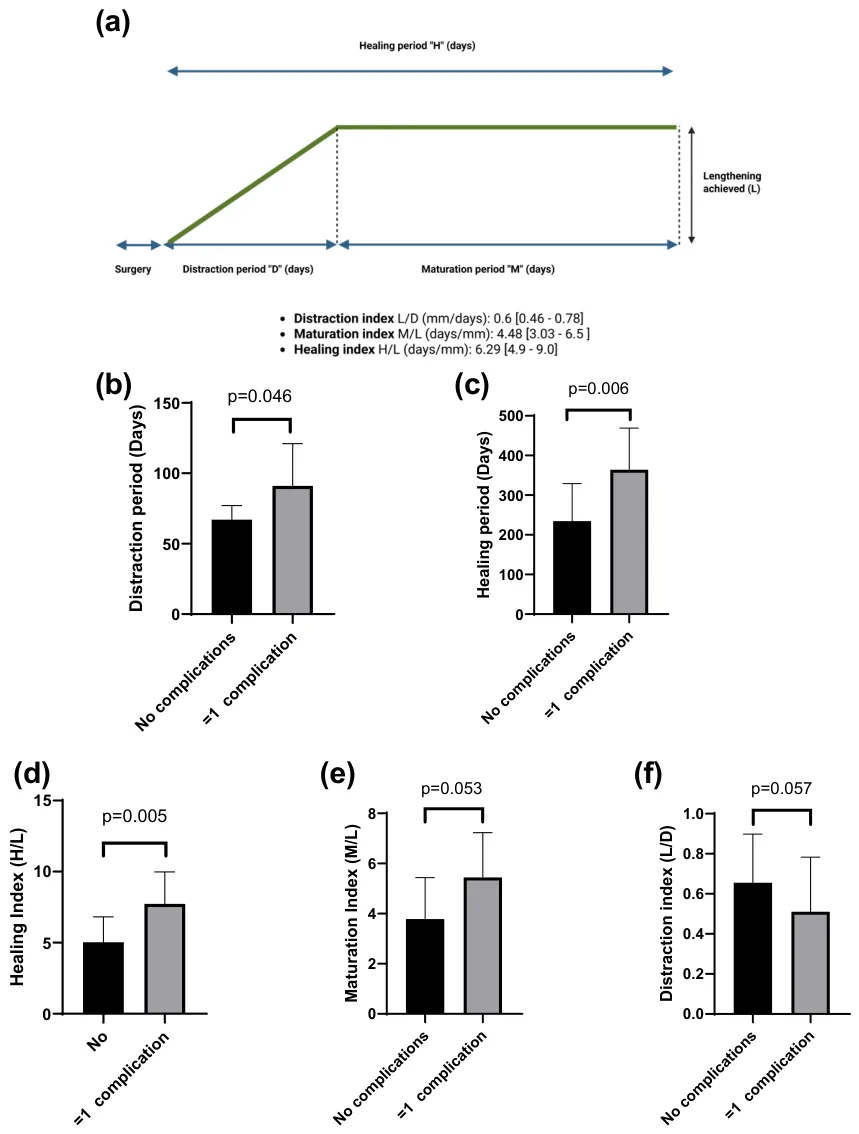
Lengthening indices and the impact of postoperative complications on treatment efficiency. (**a**) Schematic illustration of the temporal phases of distraction osteogenesis and calculation of the distraction (DI), maturation (MI), and healing (HI) indices. (**b**–**f**) Patients who experienced at least one postoperative complication required longer distraction and healing periods and exhibited higher healing indices than patients without complications, indicating a greater biological treatment burden. Although maturation and distraction indices showed similar tendencies, these differences did not reach statistical significance. Data are presented as median and interquartile range; comparisons were performed using the Mann–Whitney U test.

To objectively evaluate the efficiency of distraction osteogenesis, three standardized indices were calculated according to the temporal phases of limb lengthening (Figure 1a).

The distraction period (D) was defined as the interval from initiation of distraction until completion of the planned distraction protocol.

The maturation period (M) corresponded to the interval between completion of distraction and radiographic evidence of regenerate consolidation sufficient to permit external fixator removal.

The healing period (H) was defined as the total treatment duration with the external fixator, extending from surgery until definitive frame removal.

Achieved lengthening (L) represented the final amount of tibial length gained during distraction osteogenesis.

The following indices were calculated:

- Distraction Index (DI) = Achieved Lengthening (L)/Distraction Period (D), expressed as mm/day.
- Maturation Index (MI) = Maturation Period (M)/Achieved Lengthening (L), expressed as days/mm.
- Healing Index (HI) = Healing Period (H)/Achieved Lengthening (L), expressed as days/mm.

The distraction index reflects the average distraction rate achieved during active lengthening. Lower values indicate slower distraction or interruptions during treatment.

The maturation index reflects regenerate maturation after completion of distraction. Higher values indicate delayed regenerate consolidation.

The healing index represents overall treatment efficiency by relating total treatment duration to achieved lengthening. Higher values indicate longer treatment time required per millimeter of bone gained and therefore lower treatment efficiency.

Because distraction, maturation, and healing represent consecutive biological phases of distraction osteogenesis, these indices are mathematically interrelated rather than statistically independent variables. Consequently, each index was analyzed separately to characterize distinct phases of bone regeneration rather than as independent outcome measures (Figure 1a).

### 2.5. Complication Assessment

Complications were documented contemporaneously in the clinical records during treatment and were retrospectively reviewed for the present study.

Complications included pin-site complications, joint stiffness, premature consolidation, regenerate fracture, residual angular deformity, and other treatment-related adverse events.

For exploratory analyses, complications were evaluated using three complementary approaches:

1. Overall complication occurrence (absence/presence of any complication);
2. Complication burden, defined as the number of distinct complication categories experienced by each patient;
3. Pin-site complication severity, classified as no pin-site complication, soft-tissue inflammation, or soft-tissue infection.

This complementary classification was intended to characterize both the cumulative burden and the clinical severity of postoperative complications in relation to treatment efficiency.

### 2.6. Radiographic Assessment

Mechanical axis alignment was evaluated using standardized long-standing radiographs before treatment and during follow-up.

Radiographic measurements included planned tibial lengthening, achieved lengthening, regenerate consolidation, and alignment correction. Fractures, angular deformities, or other radiographic complications occurring during distraction or after external fixator removal were also recorded. A detailed schematic of the measurement workflow is provided in Supplementary Figure S1.

### 2.7. Statistical Analysis

Continuous variables were assessed for normality using the Shapiro–Wilk test. Normally distributed variables are presented as mean ± standard deviation, whereas non-normally distributed variables are summarized as median and interquartile range (IQR). Categorical variables are reported as frequencies and percentages.

Two-group comparisons of continuous variables were performed using the Mann–Whitney U test, and categorical variables were compared using Fisher’s exact test.

Comparisons across three or more groups were performed using the Kruskal–Wallis test.

Associations between continuous or ordinal variables were explored using Spearman’s rank correlation coefficient.

Exploratory logistic regression models were constructed to evaluate the association between planned lengthening and failure to achieve the planned goal, first in an unadjusted model and subsequently after adjustment for overall complication status.

Treatment efficiency was further explored using robust linear regression models with log-transformed healing index as the dependent variable. Regression coefficients were additionally expressed as percentage changes to facilitate clinical interpretation.

Because these regression analyses were exploratory and the cohort size was limited, they were considered hypothesis-generating rather than confirmatory.

To reduce the probability of false-positive findings arising from multiple exploratory comparisons, Holm-adjusted and Benjamini–Hochberg false discovery rate (FDR) adjusted *p* values were additionally reported where appropriate.

All statistical tests were two-sided, and a *p* value < 0.05 was considered statistically significant. Statistical analyses were performed using, IBM SPSS Statistics (Version 26; IBM Corp., Armonk, NY, USA) and R statistical software version 4.5.2 (R Foundation for Statistical Computing, Vienna, Austria).

## 3. Results

Thirty-nine pediatric patients underwent tibial lengthening using a monolateral external fixator during the study period. The median age at first surgery was 14 years (interquartile range [IQR], 11–16 years), and most patients were male (69.2%, *n* = 27). The median body mass index (BMI) was 21.3 kg/m^2^ (IQR, 17.8–25.4), median weight was 54 kg (IQR, 41.6–64.4), and median height was 1.58 m (IQR, 1.48–1.63).

The median distraction, maturation, and healing indices were 0.60 mm/day (IQR, 0.46–0.78), 4.48 days/mm (IQR, 3.03–6.50), and 6.29 days/mm (IQR, 4.90–9.00), respectively. Femoral hypoplasia associated with peroneal hemimelia represented the most frequent underlying diagnosis (46.2%), followed by isolated peroneal hemimelia (33.3%). Soft-tissue infection was the most common postoperative complication (35.9%), followed by muscle stiffness (17.9%), premature consolidation (10.3%), and regenerate fracture (7.7%) (Table 1). Representative radiographic documentation of tibial lengthening using the monolateral external fixator is presented in Supplementary Figure S2.

### 3.1. Surgical Outcomes and Goal Achievement

Overall, most patients successfully achieved the planned lengthening target. The distribution of the underlying etiologies was comparable between patients who achieved and those who did not achieve the planned correction, with femoral hypoplasia associated with peroneal hemimelia and isolated peroneal hemimelia representing the most frequent diagnoses in both groups (Table 2).

To further characterize surgical performance, planned and achieved lengthening, percentage of planned lengthening achieved, and lengthening shortfall were compared between groups (Supplementary Table S1A,B). Patients who failed to achieve the planned correction had significantly greater planned lengthening targets, lower percentages of planned lengthening achieved, and greater absolute lengthening shortfall than patients who successfully achieved their planned correction.

Correlation analyses further supported these findings (Supplementary Table S4). Greater planned lengthening was associated with lower percentages of achieved correction and greater lengthening shortfall, indicating progressively lower probability of complete correction as surgical lengthening targets increased.

Exploratory logistic regression analysis confirmed this association, demonstrating that each additional millimeter of planned lengthening was associated with higher odds of failure to achieve the planned goal (adjusted OR, approximately 1.07; 95% CI, approximately 1.01–1.16; *p* = 0.046). The presence of postoperative complications did not independently predict failure after adjustment (Supplementary Table S5).

### 3.2. Individual Complications and Treatment Efficiency

To investigate factors associated with treatment efficiency, distraction, maturation, and healing indices were compared across clinically relevant patient subgroups (Figure 1).

Female patients demonstrated significantly higher maturation indices than male patients (*p* = 0.036). Patients who developed premature consolidation had significantly higher BMI values than those without premature consolidation (*p* = 0.015).

Among all postoperative complications, soft-tissue infection demonstrated the strongest relationship with treatment dynamics. Patients with soft-tissue infection experienced significantly longer distraction periods (*p* = 0.003) and healing periods (*p* = 0.003), together with significantly lower distraction indices (*p* = 0.010) and higher healing indices (*p* = 0.002), indicating lower overall treatment efficiency compared with patients who did not develop soft-tissue infection (Figure 1).

### 3.3. Overall Complication Burden

**Figure 2:**
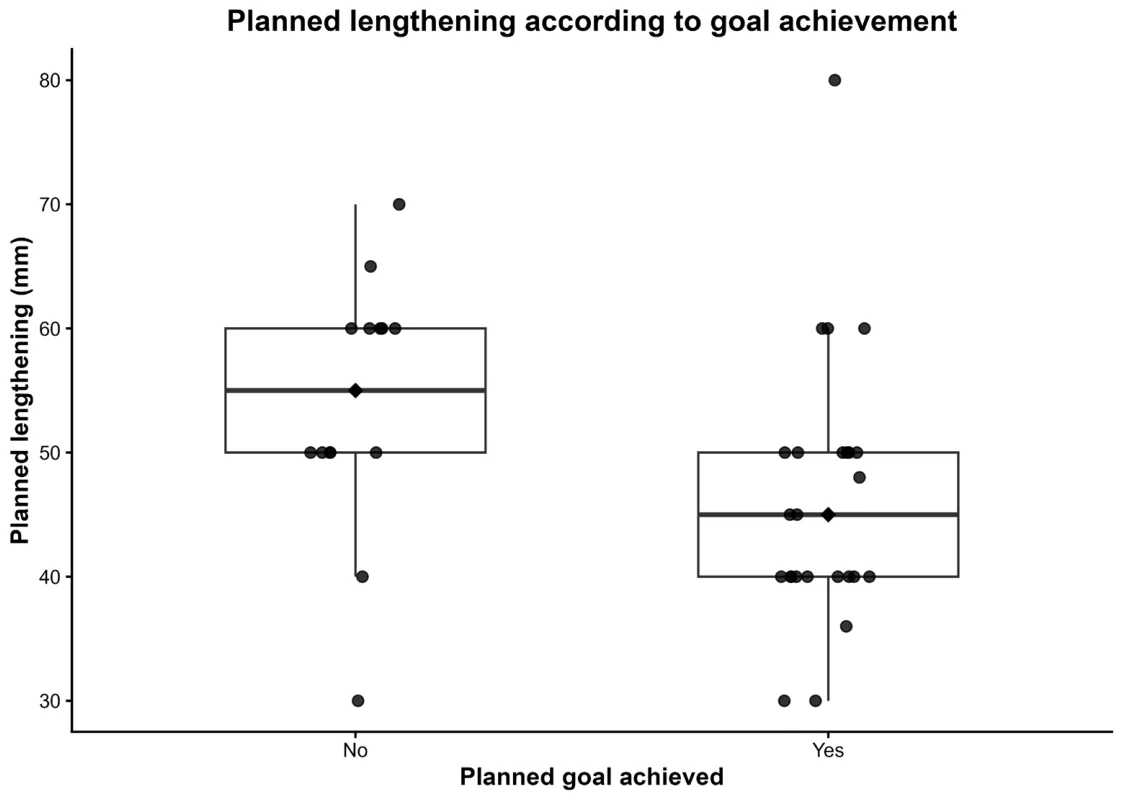
Planned lengthening according to achievement of the planned correction. Patients who did not achieve the planned correction had significantly greater planned lengthening than those who achieved the intended target. This finding supports the association between increasing surgical ambition and a lower probability of complete correction observed in the present cohort. Boxes represent the interquartile range (IQR), the horizontal line indicates the median, whiskers represent 1.5 × IQR, dots correspond to individual patients, and diamonds indicate the mean. Statistical comparisons were performed using the Mann–Whitney U test.

To evaluate the cumulative impact of postoperative complications, patients were subsequently categorized according to the presence or absence of any complication (Figure 2).

Patients who experienced at least one complication showed significantly longer distraction periods (*p* = 0.046), prolonged healing periods (*p* = 0.006), and higher healing indices (*p* = 0.005) than patients without complications. Although maturation and distraction indices also tended to differ between groups, these differences did not reach statistical significance (Figure 2).

To further explore whether treatment efficiency progressively worsened as postoperative morbidity increased, complication burden was analyzed according to the number of complication categories experienced by each patient (Supplementary Table S2A–C). A progressive increase in healing period and healing index was observed with increasing complication burden, supporting a cumulative relationship between postoperative morbidity and treatment duration.

Similarly, analyses based on pin-site complication severity demonstrated progressively longer healing periods and higher healing indices from patients without pin-site complications to those with soft-tissue inflammation and soft-tissue infection (Supplementary Table S3A–C), suggesting that increasing pin-site severity was associated with progressively lower treatment efficiency.

### 3.4. Exploratory Correlation and Multivariable Analyses

Spearman correlation analyses demonstrated consistent associations between planned lengthening, treatment duration, and lengthening efficiency (Supplementary Table S4). Greater planned lengthening correlated with lower percentages of achieved correction and greater lengthening shortfall, whereas longer healing periods were strongly associated with higher healing indices.

Finally, exploratory multivariable analyses were performed to evaluate the independent contribution of clinically relevant variables. Logistic regression identified planned lengthening as the principal factor associated with successful achievement of the planned correction, whereas robust linear regression demonstrated that postoperative complications, particularly increasing pin-site complication severity, were independently associated with prolonged healing dynamics after adjustment for achieved lengthening (Supplementary Tables S5 and S6).

## 4. Discussion

The present study provides new evidence that treatment success following pediatric tibial lengthening with monolateral external fixation should not be interpreted exclusively according to the final amount of length achieved. Instead, our findings suggest that treatment outcome may be understood through two complementary dimensions: successful surgical planning and biological treatment efficiency. Greater planned lengthening was consistently associated with a lower probability of achieving the intended correction, whereas postoperative complications, particularly pin-site complications, predominantly influenced treatment efficiency by prolonging distraction and healing periods, increasing the healing index, and extending the overall duration of treatment. Furthermore, increasing complication burden demonstrated a progressive relationship with healing dynamics, supporting the concept that cumulative postoperative morbidity increases the biological cost required to achieve successful limb reconstruction. Although these observations require prospective validation, they provide a conceptual framework that integrates technical success with the biological response throughout distraction osteogenesis [4,5,6,14].

One of the most relevant findings of this study was the association between greater planned lengthening and a lower probability of achieving the intended surgical correction. While previous pediatric series have generally reported excellent overall success rates for distraction osteogenesis, relatively few have examined which factors predict incomplete achievement of the planned correction. Most published studies have focused on describing final lengthening, complication rates, or healing indices rather than evaluating the contribution of preoperative planning itself to treatment success [5,19,20]. Our results therefore complement rather than contradict the existing literature by suggesting that the magnitude of the planned correction represents an important determinant of technical success.

This observation is biologically plausible. Increasing planned lengthening inevitably exposes the regenerate, surrounding soft tissues, neurovascular structures, and rehabilitation process to greater mechanical and biological demands. Longer distraction distances require prolonged external fixation, increase cumulative exposure to complications, and may exceed the adaptive capacity of bone and soft tissues in selected patients [4,5,6,14]. Although causality cannot be inferred from the present retrospective cohort, the consistency of this association across group comparisons, correlation analyses, and exploratory logistic regression strengthens the hypothesis that surgical planning itself contributes substantially to treatment success.

The second major finding of this study concerns the biological consequences of postoperative complications. Although most patients ultimately achieved satisfactory lengthening, those who experienced complications required significantly longer distraction and healing periods, resulting in higher healing indices and therefore lower treatment efficiency. These observations indicate that postoperative complications predominantly affect the biological course of distraction osteogenesis rather than the final amount of correction obtained. In other words, complications appear to increase the biological effort necessary to achieve successful reconstruction rather than simply preventing successful lengthening [4,17,21].

Previous investigations have consistently identified complications as an inherent component of distraction osteogenesis. Classical work by Paley introduced the distinction between problems, obstacles, and true complications and emphasized that adverse events frequently accompany prolonged external fixation [4]. More recent pediatric series similarly report pin-site infection, joint stiffness, regenerate abnormalities, and premature consolidation among the most frequent postoperative events [5,16,17]. However, most of these studies describe complication frequency without examining how postoperative morbidity influences treatment efficiency. Our findings extend this body of evidence by suggesting that complications should not be interpreted solely as safety outcomes but also as determinants of the biological performance of distraction osteogenesis.

The additional analyses performed in this study further strengthen this interpretation. Rather than evaluating complications only as present or absent, we quantified cumulative complication burden and observed a progressive increase in healing period and healing index as the number of complication categories increased. Although previous reports have recognized that multiple adverse events frequently require additional interventions throughout treatment, few studies have evaluated this cumulative effect quantitatively [4,5]. Our findings therefore suggest the existence of a biological dose–response relationship in which increasing postoperative morbidity progressively reduces treatment efficiency. This concept may help explain why patients achieving similar final lengthening often experience substantially different treatment durations and rehabilitation requirements despite comparable radiographic outcomes.

Finally, pin-site complications deserve particular attention because they represented the most frequent postoperative event and demonstrated the strongest relationship with treatment efficiency. Increasing pin-site severity was consistently associated with prolonged healing dynamics, and this association remained evident in exploratory multivariable analyses after accounting for achieved lengthening. Although superficial pin-site reactions are generally expected during prolonged external fixation and are usually managed successfully with local care, progression toward deeper infection may compromise regenerate biology, delay consolidation, and substantially prolong external fixation time [4,17]. These findings reinforce the importance of standardized pin-site surveillance and early intervention, suggesting that prevention and prompt management of pin-site complications may represent one of the most readily modifiable strategies for improving treatment efficiency during pediatric tibial lengthening.

The present findings are broadly consistent with the current understanding of distraction osteogenesis while extending several aspects that have received comparatively limited attention in previous pediatric series. Classical studies established the biological principles governing distraction osteogenesis and demonstrated that successful limb reconstruction depends on an appropriate balance between mechanical stability, preservation of vascularity, gradual distraction, and regenerate biology [4,6,14]. More recent investigations have emphasized complication profiles, healing indices, external fixation duration, and comparisons among monolateral, circular, hexapod, and intramedullary techniques [5,8,18,22]. Our results complement this evidence by suggesting that treatment success should be interpreted through two complementary dimensions: technical achievement of the planned correction and biological efficiency of the regenerative process. Within this framework, planned lengthening primarily influences the probability of reaching the intended correction, whereas postoperative complications predominantly determine the biological burden required to achieve that correction.

An additional contribution of this study is the incorporation of complication burden and pin-site complication severity as complementary indicators of treatment efficiency. Previous reports have generally evaluated complications individually or as dichotomous outcomes [4,17], whereas our analyses demonstrated a progressive relationship between increasing postoperative morbidity and prolonged healing dynamics. Although these findings remain exploratory, they suggest that cumulative complication burden may provide clinically meaningful information beyond the simple occurrence of adverse events and may help explain part of the variability observed among patients undergoing distraction osteogenesis.

This study has several strengths. All patients were treated at a single tertiary pediatric referral center using a standardized surgical strategy and postoperative follow-up protocol, minimizing variability in clinical management. Furthermore, treatment efficiency was evaluated through complementary indices representing distinct biological phases of distraction osteogenesis, while additional analyses including complication burden, pin-site severity, correlation analyses, and exploratory multivariable models provided convergent evidence supporting the principal findings. The consistency observed across these analytical approaches increases confidence in the internal validity of the reported associations.

Several limitations should also be acknowledged. The retrospective design may have introduced selection bias and limited control of unmeasured confounding variables. The relatively small sample size reduced statistical power and restricted the complexity of multivariable adjustment; therefore, the regression models should be interpreted as exploratory and hypothesis-generating. In addition, complications were not prospectively classified using standardized severity systems, and the absence of a comparison group precludes direct conclusions regarding the relative performance of monolateral external fixation versus alternative limb-lengthening techniques. Consequently, external validation in larger prospective multicenter cohorts remains necessary.

From a clinical perspective, these findings suggest that optimization of pediatric tibial lengthening should not rely exclusively on achieving the planned correction. Equally important is minimizing the biological burden required to obtain that correction. Careful preoperative planning, realistic lengthening targets, meticulous pin-site surveillance, early recognition of postoperative complications, and systematic monitoring of distraction, maturation, and healing indices may collectively reduce treatment duration while preserving satisfactory reconstructive outcomes. Future prospective multicenter studies incorporating standardized complication classifications and adequately powered multivariable prediction models will be essential to validate these observations and refine individualized treatment strategies.

## 5. Conclusions

In conclusion, pediatric tibial lengthening using monolateral external fixation may be understood through two complementary dimensions of treatment success. Greater planned lengthening was associated with a lower probability of achieving the intended correction, whereas postoperative complications, particularly increasing pin-site complication severity, were associated with reduced biological treatment efficiency through prolonged distraction osteogenesis and higher healing indices. These findings suggest that successful limb reconstruction should be evaluated not only according to the final amount of length achieved but also according to the biological cost required to obtain that correction. Although prospective validation is warranted, integrating surgical planning with strategies aimed at minimizing postoperative morbidity may represent complementary strategies for improving treatment efficiency and reducing treatment burden in pediatric distraction osteogenesis.

## Figures and Tables

**Table 1:** Patients’ characterization.

	Patients (*n* = 39)
Age at first surgery in years	14 (11–16)
Male sex, *n* (%)	27 (69.2)
BMI at first surgery	21.3 (17.8–25.4)
Weight at first surgery, “kg”	54 (41.6–64.4)
Height at first surgery, “mts”	1.58 (1.48–1.63)
Maturation index, “days/mm”	4.48 (3.03–6.5)
Distraction index, “mm/days”	0.6 (0.46–0.78)
Healing index, “days/mm”	6.29 (4.9–9.0)
Etiology, *n* (%)	
Peroneal hemimelia	13 (33.3)
Tibial Hemimelia	3 (7.7)
Femoral hypoplasia plus peroneal hemimelia	18 (46.2)
Tibial pseudoarthrosis	2 (5.1)
Femoral hypoplasia plus tibial hemimelia	2 (5.1)
Traumatic	1 (2.6)
Complications, *n* (%)	
Muscle stiffness	7 (17.9)
Premature consolidation	4 (10.3)
Bone callus fracture	3 (7.7)
Soft-tissue infection	14 (35.9)

Quantitative variables are represented as median and interquartile range, while qualitative variables are represented as frequencies and percentages. BMI: Body Mass Index, IQR: Interquartile Range.

**Table 2:** Clinical characteristics and treatment outcomes according to achievement of the planned lengthening goal.

Variable	Goal Not Achieved (*n* = 14)	Goal Achieved (*n* = 25)	Effect Size	*p* Value
Baseline characteristics				
Age at first surgery (years)	14.0 (12.0–15.0)	13.0 (11.0–16.0)	r = −0.09	0.647
Body mass index (kg/m^2^)	20.18 (17.41–24.76)	21.33 (19.07–25.37)	r = −0.13	0.529
Initial limb length discrepancy (mm)	52.5 (50.0–60.0)	48.0 (40.0–60.0)	r = 0.31	0.111
Lengthening procedure				
Planned lengthening (mm)	55.0 (50.0–60.0)	45.0 (40.0–50.0)	r = 0.48	0.012
Achieved lengthening (mm)	49.0 (40.5–56.3)	48.0 (42.0–53.0)	r = 0.03	0.895
Distraction period (days)	77.0 (69.3–105.5)	70.0 (56.0–98.0)	r = 0.12	0.538
Maturation period (days)	199.5 (143.3–279.0)	218.0 (175.0–294.0)	r = −0.12	0.548
Healing period (days)	307.0 (198.0–362.8)	301.0 (239.0–385.0)	r = −0.18	0.372
Healing index (days/mm)	6.09 (4.43–9.29)	6.68 (5.11–8.54)	r = −0.11	0.588
Complications				
Any complication, *n* (%)	9 (64.3%)	16 (64.0%)	V = 0.003	1
Number of complication categories	1 (0–1.75)	1 (0–2.0)	r = 0.01	0.963

Data are presented as median (interquartile range) or number (percentage), as appropriate. Continuous variables were compared using the Mann–Whitney U test and categorical variables using Fisher’s exact test. Rank-biserial correlation (r) and Cramer’s V (V) are reported as effect-size measures.

## Data Availability

Raw data may be shared as a reasonable request to corresponding authors.

## Supplementary Materials

The following supporting information can be downloaded at: https://doi.org/10.5281/zenodo.21362062 accessed on 15 July 2026.

## Abbreviations

The following abbreviations are used in this manuscript:

BMI	Body Mass Index
IQR	Interquartile Range
LLD	Lower Limb Length Discrepancy
AP	Anteroposterior
mm	Millimeter
cm	Centimeter
kg	Kilogram
m	Meter
